# Case of Pelvic Abscess Opened through the Rectum

**Published:** 1846-08

**Authors:** 


*Case of Pelvic Abscess opened through the Rectum.* By M. Amussat. Related by Dr. Compérat.—The subject of the case was a lady, 37 years of age, who had borne two children. She had enjoyed excellent health until 10 years since, when her husband infected her with gonorrhœa, from the secondary effects of which she suffered and recovered; a severe lumbar pain, however, then making its appearance, and continuing to annoy her at intervals. During the last 18 months this pain had increased in severity, and when first seen, 4th April, 1844, by Dr. Compérat, it had become very intense, occupying all the right side of the abdomen and genital organs. We need not pursue the detail of her sufferings, and of the measures which partially relieved them; but may observe that, by a recto-vaginal examination, a tumefaction was felt on the posterior part of the right side of the body of the uterus, seeming to be seated exactly at the recto-vaginal partition. Passing the index-finger high up into the rectum, its pulp was just able to reach the projecting part of another large swelling at the right posterior portion of the gut. All the parts were hot and irritable, and the examination gave excruciating pain. MM. Amussat and Fouquier saw the case, and believed it to be one of abscess, which the great constitutional irritation would seem to indicate, although they could not detect fluctuation, which Dr. C. believed he had felt. As the point of the finger could only just reach the swelling, the surgeons wished to defer opening it, and ordered cataplasms, the simultaneous injection of the rectum and vagina with emollients, and other palliatives. On the 19th, fluctuation had become distinct, and as the patient's general condition was now truly alarming, they determined to operate. M. Amussat having passed his finger into the rectum as high as the fluctuating tumour, slid along its palmar aspect a pair of very sharp-pointed scissors, very like those contained in dissecting cases, but having the backs of the blades rounded and the branches very much longer. The puncture was made and the blades then separated, so as to enlarge the aperture by tearing rather than cutting. During this period, an assistant pressed upon the belly, so as to project the tumour as far towards the rectum as possible. A lithotrity catheter was now substituted for the scissors, and guided along the finger, still left in the rectum. A considerable quantity of healthy, and scarcely sanguinolent pus flowed out. The cavity of the abscess was carefully injected with tepid water, and the rectum cleaned out, and the access of air prevented by ascending douches of the same. These last gave great relief. In order to keep the aperture of the abscess patent, the index-finger was introduced several times during the first day. The next day, however, it had nearly closed, and the finger could not pass through the thickness of the walls of the abscess, which M. Amussat had observed seemed like cutting into the substance of the uterus itself. He enlarged the opening by means of a longer and stronger pair of scissors, the blades of which were notched near their extremities, so as to give the instrument a lance form, and prevent its escaping during the attempt at enlarging the wound. The edges were kept separate by occasionally expanding them by means of a *brise-pierre*. The case eventually did very well.

Dr. Compérat observes that, although numerous cases of abscess forming in the cavity of the pelvis are on record, (M. Bourdon gives an account of 23 of these in his paper "on fluctuating abscesses of the pelvis, and their discharge by an aperture practised in the vagina," in the *Rev. Medicale*, 1841,) yet the present, as far as he knows, is the first case in which an opening has been made for the evacuation of the pus by the rectum. The surgical procedure in each case must depend upon the direction which the projecting portion of the swelling takes. Of course, the difficulties to be surmounted are less in the vaginal incision. One of these, the danger of *hæmorrhage*, was met in this case by using very pointed scissors, first as a trocar, and then tearing, rather than cutting, the parts with them. The edges of the aperture also were effectually dilated by means of the *brise-pierre* as long as requisite. The introduction of instruments, however, at last induced severe *irritability of the anus*, which continued for hours, and caused the patient to dread its repetition beyond everything. M. *Recamier*, who saw the case, promised her, if she could bear very severe pain for a short period, to affectually relieve her.

"Having well oiled his fingers and thumb he formed them into the shape of a cone, and thrust them altogether through the anal ring, impressing upon the entire hand slight movements of supination and pronation in order to facilitate its introduction. The margin passed, the fingers were bent so as to increase the size of the imprisoned mass, and then drawing them out at once in this position, he forcibly dilated the sphincter, thereby causing excessive pain for some minutes. The anus spasmodically contracted during several hours, and then all became quiet. From this period, the sphincter offered no obstacle to the introduction of the finger and *brise-pierre*, and the patient suffered no pain whatever from it. I do not know whether M. R. has published his ingenious expedient; but all I can say is that, from the surprising manner in which it relieved the excessive sensibility of the anus and the anguish of the patient, it cannot be too strongly recommended in all cases where the permanent or frequent introduction of instruments irritates the sphincter."—*Med. Chir. Rev. from Revue Medicale.*

